# The Mechanical Genius and the Pen

**Published:** 1919-04-26

**Authors:** 


### THE MECHANICAL GENIUS AND THE PEN.

A correspondent writes : I have a mechanical genius. A bit of mechanism attracts me as a lighted candle attracts a moth. Whenever a clock in the house stops, I take it to pieces, clean it, put it together again; and after this operation it sometimes goes. When the gas burns dim, I take the burners to pieces and clean them, and sometimes I succeed in doing this without breaking the globe. A screw has a fascination for me, and I can never see a thing screwed together but I want to take out the screws and put them in again, and this propensity has led me into disaster. Yesterday I became the happy possessor of a fountain pen. I hastened to take it out of its box and examine first its graceful proportions, and then its mechanical construction. I unscrewed the point section and screwed it on again, not without a suspicion that the pen had been used, for there seemed to be traces of ink on it. Then I unscrewed the other end, and pulled on it so as to draw out the piston. It certainly seemed to be beautifully made, for the piston fitted to perfection. It glided with fascinating smoothness in the barrel of the pen, but it opposed more resistance than I had expected. Having satisfied myself that the piston-rod was well finished and the pen in all respects well made, I gently pressed the piston back into its place and screwed the butt of the pen on to the shaft.

The consequence was horrible. A fountain of ink burst from the nib of the pen, and smothered everything within reach. This is, no doubt, the reason, and an excellent reason, why the maker gives to it the title of a fountain pen. I should call it a cloud-burst pen, or a deluge pen. The ink covered my hands; it blotted the paper on which I was writing; it smothered my clothes, paying particular attention to my shirt cuffs; it filled the seat of my chair, ran down into my shoes, overflowed upon the floor. It ran in a black stream across the floor, under the door, and presently I heard it dribbling from stair to stair. "Who would have thought" says Lady Macbeth, "the old man to have so much blood in him?" Who would have thought, say I, that one little pen could contain gallons and gallons of ink? I expect to hear that it has overflowed into the street, and that passers-by have been swept away by the flood.

When the mess was sufficiently cleared up, I perused the literature that is supplied with the pen, and found that the purchaser is warned that the pen is full of ink, and that he must not unscrew the part marked "B." But what is the use of a warning that is not given until after the disaster has happened? My papers are spoilt, the book I was referring to is spoilt, my clothes are spoilt, and my hands . . . fortunately this catastrophe happened on a Friday, and my day for washing my hands is Saturday, so that I had only one day of disfigurement; but it would have been very awkward if the pen had arrived on a Monday. I am still finding blots and pools of ink in unexpected places. Like a notorious character of Mr. Rudyard Kipling's, I am constrained to say the damn-damns.

